# The Emergent Engram: A Historical Legacy and Contemporary Discovery

**DOI:** 10.3389/fnbeh.2018.00168

**Published:** 2018-08-07

**Authors:** Bryan D. Devan, Kyle Berger, Robert J. McDonald

**Affiliations:** ^1^Laboratory of Comparative Neuropsychology, Psychology Department, Towson University, Towson, MD, United States; ^2^Canadian Center for Behavioural Neuroscience, University of Lethbridge, Lethbridge, AB, Canada

**Keywords:** memory, memory trace, history, technology, cell assembly, functional circuits, engram, subcortical-cortical connectivity

## Overview

Technological advances have made it possible to capture a specific assembly of neurons active during a learning event and manipulate the captured cells, demonstrating some form of relationship between brief retention events and the cell assembly. The reductionists' claim of localizing the engram has been met with considerable skepticism. The potential for off-target effects in “cell capture” techniques, such as optogenetics, reveal serious limitations when given the highly interconnected nature of brain networks beyond a selective assembly of cells in a restricted area of manipulation. Recent studies of subcortical-cortical interactions prompted us to review the historic search for the engram, revealing a parallel between the empirical attempts to localize the engram in cortical then subcortical systems. When brought together conceptually, the very extensive independent work of Karl Lashley and Robert Thompson suggest that each held a piece of the puzzle. Recent research implicates the interaction between subcortical memory systems and association cortex in the transformation of mnemonic information from a short-term process of plasticity to a long-term state of stability. We propose that one function of subcortical-cortical connectivity is to continuously update contextually retrieved long-term memory during reconsolidation of newly acquired information processing, creating a constantly evolving emergent engram.

## The early engram

Researchers claim to now “capture” activated cells allocated during a learning experience to represent an enduring change among an assembly of neurons called an engram (Josselyn et al., 2015; Tonegawa et al., 2015). Semon (1921, p. 12) made up the term and defined it as “…the enduring though primarily latent modification in the irritable substance produced by a stimulus.” Semon favored his novel word creation to avoid undesirable connotations of everyday terms in favor of a precise scientific definition. However, he did not provide a precise definition due to “a lack of physiological knowledge at the time,” and claiming that “…such speculation was unwarranted” (Schacter et al., 1978, p. 726). The lack of a satisfactory definition has led to the “filling-in” of what's missing scientifically, and has become popular in mainstream culture (from scientology to popular video games).

## Two routes of investigation

Research on the engram then took two routes of inquiry. The first route (Hebb, 1949), described cell assemblies and the “learning rule” as an enduring synaptic change later supported by the discovery of long-term potentiation (Bliss and Lomo, 1973; Bliss and Collingridge, 1993). The second route, taken by Hebb's mentor, Karl Lashley (Lashley, 1950; Bruce, 2001), set out to localize an engram empirically somewhere within association cortex, thought to be the repository of long-term memory (Battaglia et al., 2004; Dash et al., 2004; Rauchs et al., 2005; Girardeau et al., 2009; Wang and Morris, 2010; Insel and Takehara-Nishiuchi, 2013; Wiltgen and Tanaka, 2013; de Voogd et al., 2016; Sekeres et al., 2018).

Karl Lashley used various post-training lesion techniques to disrupt the retrieval of memory in rats and monkeys (Lashley, 1950). After decades of systematic empirical work, Lashley concluded that it was not possible to localize an engram in association cortex because memory was widely distributed throughout a network of neurons that all have an equal potential to contribute to the memory trace. Robert Thompson, continued Lashley's work in subcortical structures using pre- and post-training lesions (Thorne, 1995), and published two books (Thompson, 1978; Thompson et al., 1990) that summarized findings from over 120 published journal articles.

Though his work has not been widely recognized in recent reviews of the engram (Josselyn et al., 2015; Tonegawa et al., 2015), it has been pointed out that Thompson was friends with Karl Lashley at the Yerkes Primate Center where they discussed research and the lack of progress in localizing an engram (Thorne, 1995). Robert Thompson thought that Lashley may not be looking in the right place for engrams and, his interest in Penfield's Centrencephalic Integrating System (Penfield, 1958), as the general learning system (Thompson, 1993), resulted in his continuation of a systematic empirical search within subcortical structures, resulting in greater success than Lashley's work (Thorne, 1995).

The success of Thompson's work supports the more modern research of parallel and interactive memory systems in subcortical structures (McDonald et al., 2017). Thompson declared that a level of localization was possible with pre-training lesions among subcortical substrates, later borne out by findings as the understanding and sophistication of behavioral tests were designed for associative learning specificity (Packard et al., 1989; McDonald and White, 1993, 2013; White et al., 2013). More recently, studies of cooperative interactions between memory systems and memory subsystems using the water maze paradigm in rats (Devan et al., 1996, 1999; Devan and White, 1999) provide new models of cognitive-habit interactions (Devan et al., 2011, 2016; Sukumar et al., 2012).

Scientific advancement using the latest technology in localizing engrams depends on the systems-level circuit analysis of functional interactions. First proposed by Richard Hirsh (Hirsh, 1974; Hirsh and Krajden, 1982) and later exemplified by the meticulous, cell recording, lesion and chemical inactivation work by Richard F. Thompson and colleagues (The “R. Thompson” ambiguity may have contributed to the confusion and lack of recognition for Robert Thompson) identified “temporal neuronal models” of activation formed in hippocampus early in classical conditioning (Berger et al., 1976). Richard F. Thompson and colleagues then discovered the essential role of the cerebellar dentate-interpositus nuclei in learning and performance of classically conditioned skeletal responses (McCormick and Thompson, 1984). A combination of conceptual and methodological approaches continue to define memory substrates and circuits within cerebellum (Poulos and Thompson, 2015) and the general advancement of the neurobiology of learning and memory (Thompson, 1986). This kind of extraordinary scientific progress, or strong inference (Platt, 1964), depends on converging operations in eliminating alternative/competing explanations of scientific findings.

## The present state of the engram

Decades of research to localize an engram using various techniques in the past (Lashley, 1950; Thompson, 1978, 2005, 2013; Thompson et al., 1990; Mayford, 2014; Eichenbaum, 2016) has provided a foundation for the current state of engram research, expressed in a recent Forum (Poo et al., 2016). Different levels of organization of an engram were considered, including processes from chemical, synaptic, and cell levels. However, systems-level interactions were largely missing from the discussion, except for proposed changes in the site of engrams from subcortical structures to association cortex. Robert Thompson and Lashley's independent work on these very locations warrant further consideration within an interactive memory systems perspective.

**Figure 1:**
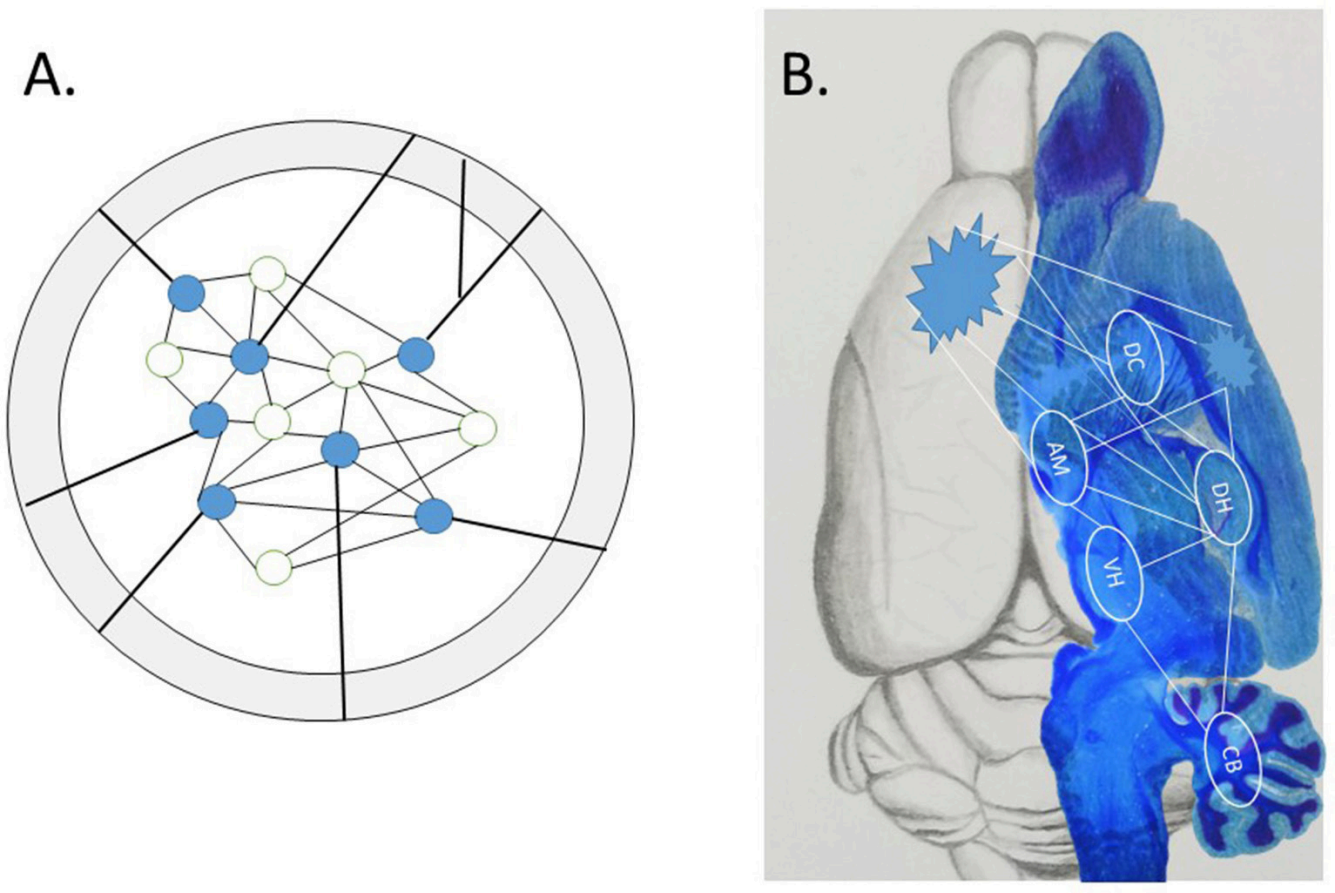
**(A)** A local engram identified by cell capture techniques within a defined brain structure. This engram is precise yet conceptually isolated from the rest of the brain. Evidence reviewed in text show “off-target” effects outside the *in vitro*-like conceptual environment, as projections from axon fibers interconnect the local engram with widespread subcortical and cortical targets. **(B)** The emergent engram with subcortical and cortical components. The wide distribution of connectivity balances the scope of local engrams (pointed clusters) and distributed components of the potential system-wide interactions among multiple subcortical memory systems, with routes to association cortex for (re)consolidation of stable long-term memory representations, thought to occur during sleep (Sara and Hars, 2006; Sara, 2017).

Two exciting papers recently published in *Science* (Khodagholy et al., 2017; Kitamura et al., 2017) begin to show how the neglected interactive systems-level is involved in the wide distribution of the engram over time and space, i.e., the succession of events in neuronal activity (Buzsáki and Llinás, 2017). As no approach is perfect, the weaknesses of one are offset by the strengths of another; such *converging operations* therefore strengthen the conclusions that could not be reached by either approach alone. Although the conceptual strength of optogenetics and other cell “capture techniques” is the precise localization of the sparse network of neurons that make up an *isolated* engram (see Figure 1), the physical representation of a complex episodic engram is much more likely the emergent property of interactive memory systems (Tulving, 1987; Nyberg et al., 1998; McDonald et al., 2004a,b, 2017). Other technical problems of optogenetics may be considered, such as off-target effects in downstream circuits, as well as other spatial and temporal factors (Otchy et al., 2015; Südhof, 2015; Yates, 2016; Hardt and Nadel, 2017; McDonald and Deibel, 2017). For example, the specificity of a few neurons active in one area seems to mimic an *in vitro*-like isolation approach, while the snapshot-like association specificity in learning limits the unfolding of an event over time. A densely interconnected brain with molecularly distinct subclasses of neurons, glia and vasculature cell types in different brain areas (Zeisel et al., 2015) may expand the simple redux engram.

The above studies illustrate widespread connections and changes in plasticity that may form the neurochemical and electrophysiological substrates of a broader episodic engram representation. In the first study (Kitamura et al., 2017), functional reorganization of circuits occurred at the systems level from hippocampal and basolateral amygdala engrams, slowly strengthening the vertical pathways to prefrontal assemblies, while the hippocampal engrams became silent and amygdala engrams remained active. In the second study (Khodagholy et al., 2017), instead of optogenetics, a NeuroGrid and subcortical recording of local field potentials and spiking throughout dorsal cortex, revealed that not only does hippocampal activity transform prefrontal activity during sleep following learning, but also ripple-ripple coupling alters parietal and midline cortical activity, possible components promoting widespread network change throughout association neocortex (Buzsáki, 1996; Xu et al., 2016). Perhaps even long-term consolidation (McGaugh, 1966) over time may continuously alternate between malleable and rigid states of change in a never-ending cycle throughout life (Dudai and Eisenberg, 2004; Dudai, 2012).

## The emergent engram

It is apparent that the isolated engram is now expanded to an interactive memory systems representation with widespread association cortex coupling (Khodagholy et al., 2017) and subcortical silencing (Kitamura et al., 2017) before recall (Figure 1). It took Lashley three decades to conclude that the cortical component was widely distributed, and several more years for Robert Thompson to begin to localize subcortical substrates. In our view, this extensive empirical and theoretical work has set the stage for important future scientific discovery. An integration among ascending vertical (subcortical-neocortical) with horizontal (cortico-cortical) connectivity (Qin et al., 1997) will further our understanding of the emerging engram. A balanced scientific approach may allow us to appreciate the complexities and vastness of the forest in relation to the details of individual trees.

## Conclusion

Although the specific term “emergent engram” does not appear in major databases (e.g., PubMed and PsychINFO), its general idea is prevalent throughout contemporary memory research. Indeed, it is prominent throughout cognitive psychology, e.g., in the writings of Endel Tulving with the emergence of multiple memory systems (Tulving, 1987; Nyberg et al., 1998) among others in the Gestalt tradition (Kałamała et al., 2017; Allon et al., 2018). In addition, it has more contemporary relevance to other recent work in behavioral neuroscience (Sara and Hars, 2006; Sara, 2017). For example, Sara (2010) eloquently summarized the view as “…memory is a dynamic property of the nervous system, in constant flux as a result of being retrieved within current cognitive environments” (pg. 4).

The recent physiological findings described above are consistent with previous studies of coordinated activity within several interactive systems. For example, associative learning of a spatial discrimination was related to fast oscillations or pronounced ripple activity in the hippocampus (Ramadan et al., 2009). Neocortical slow oscillation synchrony increased with thalamocortical spindle activity and hippocampal ripple activity during non-REM sleep (Mölle et al., 2009). These findings may relate to hippocampal-cortical/subcortical coordination of reactivation events (Skelin et al., 2018) involving multiple subcortical memory systems with densely distributed networks of “hard” synaptic changes among cells in different neocortical assemblies (Milner, 1989, 1996).

The interactions among subcortical memory systems and the coordination of spatial processing within the hippocampus and the thalamocortical output of basal ganglia loops (Alexander et al., 1986, 1990; Méndez-Couz et al., 2015) may mediate a form of cognitive-habit integration (Devan et al., 2011, 2016). This hypothesis is consistent with fMRI studies that show cooperation between these systems in the flexible navigation of virtual spatial tasks (Brown et al., 2012; Woolley et al., 2015), in the demonstration of exceptional memory performance among competitive mnemonists (Muller et al., 2018) and disrupted functional connectivity between hippocampus and caudate nucleus in patients suffering from obstructive sleep apnea (Song et al., 2018). Further evidence suggests a potential role of glia in consolidation-related sleep processes (Hyden and Lange, 1965; Chen et al., 2015; Frank, 2018).

In summary, the historical search for the engram identified ascending vertical subcortical-cortical networks supporting consolidation processes in studies using the latest technology (Khodagholy et al., 2017; Kitamura et al., 2017). Further, support for multiple memory systems provided horizontal integration among subcortical systems (Devan et al., 2011, 2016). The combination of connections enable soft or labile interactions between local subcortical cell assemblies and a dense or hard (Milner, 1989) network of vastly rich cortical associations that are constantly responsive to smaller units of reconsolidation through the different stages of sleep (Sara and Hars, 2006; Sara, 2017). As we learn more about glial cells and sleep physiology (Panatier et al., 2006; Fields, 2011; Fields et al., 2014; Cousins et al., 2016; Frank, 2018) we may gain valuable insight into the complexities of yet unknown processes supporting (re)consolidation, hence continually remolding the emerging engram to express both stability and change.

## Author contributions

BD conceived of idea talking with RM regarding a previous paper. Worked on a novel conceptualization of the engram with the other authors. KB worked on historical account, and the novel conceptualizations with other authors. RB wrote a critical commentary on a high profile journal article using the term engram and helped BD to conceptualize the new emergent engram framework.

### Conflict of interest statement

The authors declare that the research was conducted in the absence of any commercial or financial relationships that could be construed as a potential conflict of interest.
